# Factors Contributing to Higher Maternal Mortality and Preterm Birth Rates Among Black and South Asian Women Than Caucasian Women in Leicester: A Narrative Review

**DOI:** 10.7759/cureus.114857

**Published:** 2026-08-20

**Authors:** Subhaan Hafeez, Samira Mendheria

**Affiliations:** 1 Medicine and Surgery, Furness General Hospital, Barrow-in-Furness, GBR; 2 Medicine and Surgery, Leicester Royal Infirmary, Leicester, GBR

**Keywords:** afro-caribbeans, ethnic minority, leicester, maternal health, maternal morbidity. maternal death, maternal mortality or death, pre-term birth, preterm labor, south asian, south asian women

## Abstract

This narrative review aims to explore why ethnic inequalities in preterm birth and maternal mortality are present, specifically among Black and South Asian women within the United Kingdom. We are particularly focusing on Leicester and Leicestershire. This review aims to address the underlying reasons as to why these inequalities are present. Leicester has a high proportion of ethnic minorities compared with the rest of the United Kingdom, making it an important context for exploring disparities in maternity outcomes.

A structured search in Medline (OVID) was conducted using keywords and MeSH terms related to ethnicity, maternal mortality, and preterm birth. Studies conducted only within the UK were included, as no Leicester-specific studies were identified; findings were therefore considered in relation to Leicester's diverse demographic to assess the likely relevance in this setting rather than direct evidence for this population. Overall, 90 studies were screened against the inclusion criteria; 18 studies were deemed relevant; however, due to time constraints, 8 were included within this narrative review.

The findings showed consistent evidence of increased risk of preterm birth and maternal mortality within the Black and South Asian population. Socioeconomic and demographic factors were unable to explain this increased risk. Some studies did show that these ethnic minorities engaged in more positive health behaviors than Caucasian women, therefore suggesting that lifestyle and health factors are not an adequate explanation for these inequalities. Some suggested explanations for these inequalities include language barriers, limited access to care due to a lack of knowledge regarding services available, and negative experiences with the NHS. However, many of these explanations do not fully explain inequalities in ethnic minorities who were born within the UK, who have no language barriers present, and who are aware of the services available. Overall, no definitive cause was found across the studies. Also, no specific study was found in Leicester or the surrounding Leicestershire area.

This review further demonstrates that there is a substantial inequality present in preterm birth and maternal mortality within the UK; however, the exact amount of inequality within Leicester and surrounding areas is unknown due to a lack of research. Given Leicester's exceptionally high minority population, it is plausible that these inequalities may be similar or more prevalent within Leicester and Leicestershire due to the higher ethnic minorities compared with the rest of the UK; however, this remains an inference drawn from wider UK evidence rather than local evidence. This narrative review suggests that multiple complex factors could explain these inequalities; however, it is unlikely that lifestyle factors alone explain these inequalities. Further research is needed, particularly within Leicester, to understand why these inequalities are present and to provide solutions to help remedy these inequalities.

## Introduction and background

Pregnancy and childbirth are major events in a woman’s health journey. For many women, this can be complicated by new or pre-existing health concerns, which can affect her continuing health and the health and delivery of the newborn baby. 

**Figure 1:**
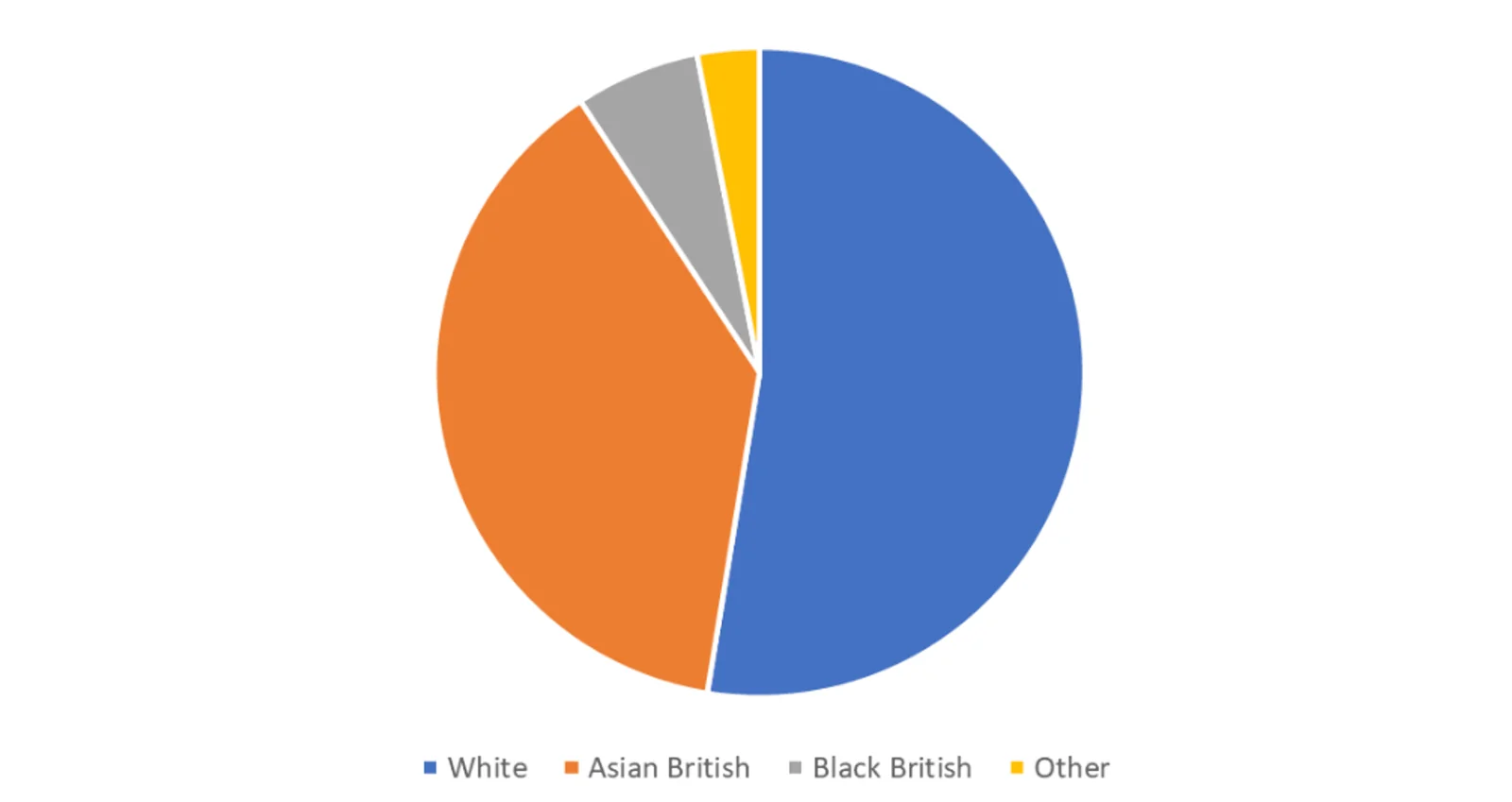
Ethnicities in Leicester [1]

**Figure 2:**
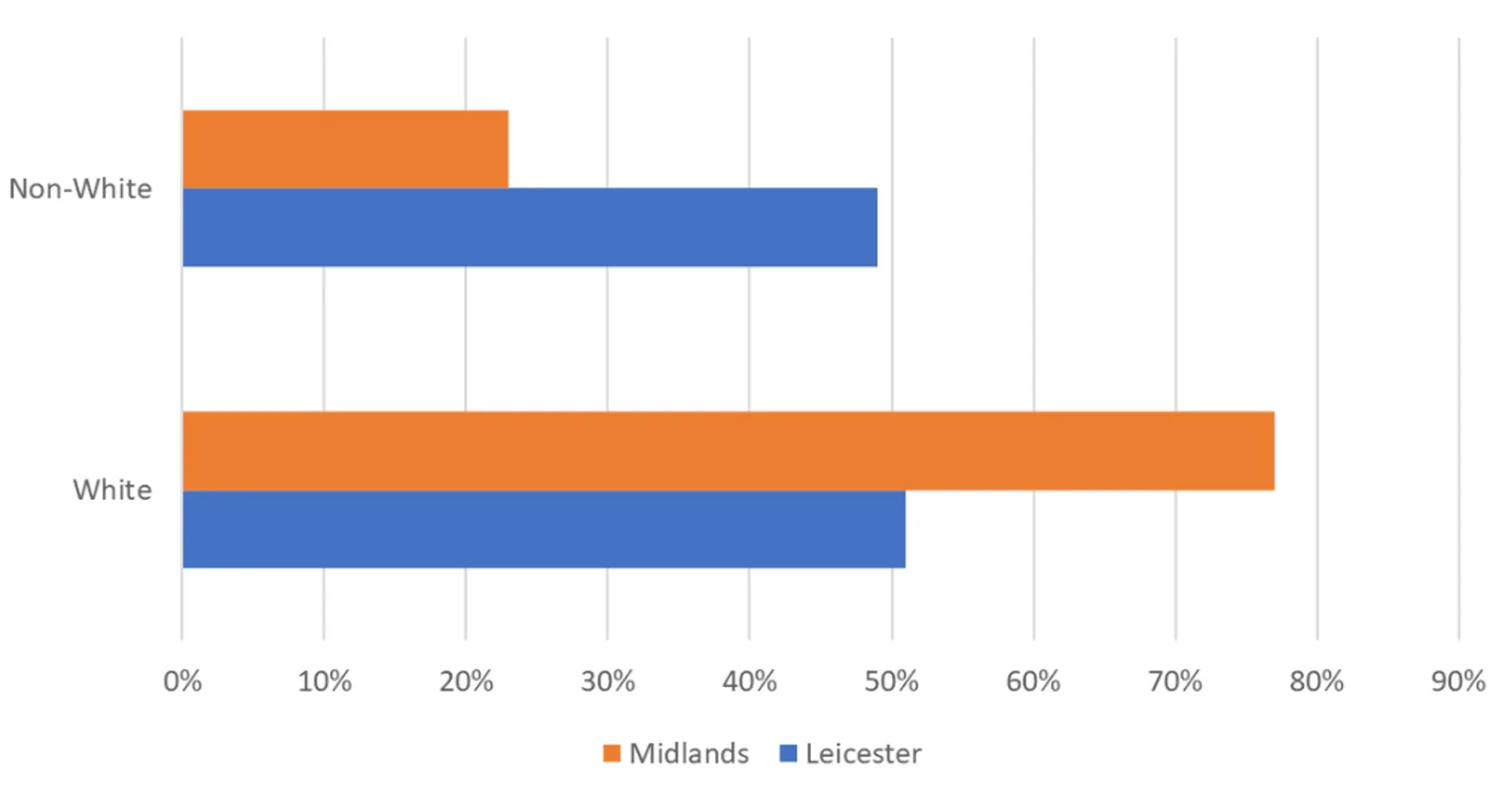
Ethnic breakdown in the midlands [1]

Leicester is a highly diverse area with a high ethnic minority population in comparison to the rest of the United Kingdom. Within Leicester, 49% of the population (Figure 1) are of an ethnic minority [1], compared with 23% (Figure 2) in the rest of the Midlands [2]. Of this 49%, 37% are Asian British, 6% are Black British, 3% are mixed, and 3% are categorized as other. Leicester and Leicestershire had a high influx of migrants after World War 2. Leicester was deemed an attractive area for migration due to cheap rents and increased work availability following an increased demand for labor [3]. Different ethnic minorities migrated at different time periods. For example, there was a high influx of South Asian migrants, specifically Pakistani and Indian, after the partition in 1947. The Caribbean population also increased, likely due to labor shortages and the need to help rebuild the infrastructure [3]. Multiple reasons and worldwide events have impacted the demographics of Leicester and surrounding areas, leading to a high percentage of ethnic minorities compared with the rest of the United Kingdom. 

A range of factors has been proposed in wider literature to explain ethnic disparities in maternal mortality and preterm birth. Some of these factors include socioeconomic deprivation, housing, stress, access and knowledge of health care services, language barriers, higher underlying prevalence of comorbidities such as gestational diabetes, and differences in quality of care received compared with their Caucasian counterparts [4]. Evidence for these factors varies in strength, where some factors, such as socioeconomic factors, are more supported than others; however, some factors, such as biological or genetic explanations for these inequalities, are more contested and are criticized for overlooking the role of other healthcare-related factors. However, the evidence remains fragmented; the current literature that does exist explores these factors individually, and no review has comprehensively brought them together within a UK context. It still remains unclear which factors are best supported by direct evidence.

A 2024 report “addressing racial disparities in maternal outcomes for the population of Leicester, Leicestershire and Rutland” showed various outcomes in ethnic groups being considerably worse, especially in Asian and Black Afro-Caribbean women, compared with Caucasian women [1]. Maternal mortality, defined as death during or up to a year after the end of pregnancy, is generally uncommon; however, there is still a clear ethnic inequality present. There is a fourfold increase in maternal mortality among women of Black ethnic background and an almost twofold increase among women of Asian ethnic background compared to White women, a disparity that has changed little since the previous reporting period [1]. In addition, premature births were shown to be higher among Black or Black British Caribbean women than other ethnicities [1].

Leicester and Leicestershire were selected as the geographic area of interest within this review, firstly due to the high and diverse ethnic minority populations documented above, and secondly due to the documented ethnic inequalities. However, our searches identified no studies examining the ethnic disparities in maternal mortality or preterm birth specific to Leicester or Leicestershire. We therefore drew upon wider UK studies and, with Leicester's demographics in mind, considered the relevance and applicability of these findings, rather than relying on a direct evidence base. Given the heterogeneity of the available evidence and the absence of any specific Leicester or Leicestershire studies, a narrative review rather than a systematic review was considered more appropriate, to allow for a broader and more flexible synthesis of the relevant literature.

This narrative review attempts to summarize evidence and explore reasons as to why Black Afro-Caribbean and South Asian women have higher rates of maternal mortality and premature births than Caucasian women in the UK and to evaluate the extent to which this evidence is applicable to Leicester. This narrative review also attempted to identify gaps that may warrant further Leicester-specific research. The location of any studies providing results or conclusions about the UK should be considered with Leicester demographics in mind.

## Review

Methodology 

Search Strategy 

We conducted a search of Medline (OVID), an electronic database that indexes journals in biomedicine, on 3 July 2023. Our search strategy used full, truncated, and MeSH terms, which included maternal mortality, preterm birth, ethnic and racial inequalities, various ethnicities (Black, Asian, Indian, Pakistani, and Afro-Caribbean), and locations (Leicester, Midlands, UK, England, London, Bradford, and Birmingham). No date limitations were applied. Reference lists were also reviewed for other potential studies. Our search was supported by an academic medical librarian and a researcher in population health sciences. Table 1 details the Medline search strategy.

**Table 1:** Medline search strategy

Search line	Search terms
1	maternal mortalit* or maternal death* or premature birth* or preterm birth* or early birth*).mp. [mp=title, book title, abstract, original title, name of substance word, subject heading word, floating sub-heading word, keyword heading word, organism supplementary concept word, protocol supplementary concept word, rare disease supplementary concept word, unique identifier, synonyms, population supplementary concept word, anatomy supplementary concept word]
2	((Ethnic or racial) adj3 (disparit* or inequal*)).mp. [mp=title, book title, abstract, original title, name of substance word, subject heading word, floating sub-heading word, keyword heading word, organism supplementary concept word, protocol supplementary concept word, rare disease supplementary concept word, unique identifier, synonyms, population supplementary concept word, anatomy supplementary concept word]
3	(black or asian or indian or pakistani or afro-caribbean or afro caribbean or afrocaribbean).mp. [mp=title, book title, abstract, original title, name of substance word, subject heading word, floating sub-heading word, keyword heading word, organism supplementary concept word, protocol supplementary concept word, rare disease supplementary concept word, unique identifier, synonyms, population supplementary concept word, anatomy supplementary concept word]
4	leicester or midlands or united kingdom or UK or London or Bradford or England or Birmingham).mp. [mp=title, book title, abstract, original title, name of substance word, subject heading word, floating sub-heading word, keyword heading word, organism supplementary concept word, protocol supplementary concept word, rare disease supplementary concept word, unique identifier, synonyms, population supplementary concept word, anatomy supplementary concept word]
5	2 or 3
6	Exp Maternal Mortality/
7	Exp Premature Birth/
8	1 or 6 or 7
9	Exp United Kingdom/
10	national health service or nhs).mp. [mp=title, book title, abstract, original title, name of substance word, subject heading word, floating sub-heading word, keyword heading word, organism supplementary concept word, protocol supplementary concept word, rare disease supplementary concept word, unique identifier, synonyms, population supplementary concept word, anatomy supplementary concept word]
11	4 or 9 or 10
12	5 and 9 and 11

We acknowledge that only one database was used, which is a limitation of this narrative review. Medline was selected, as it is a comprehensive, widely used database for clinical literature. It also has vast coverage of topics needed in this narrative review, such as maternal health and ethnicity. To mitigate the risk of missing relevant studies, our search strategy combined multiple MeSH terms, free text keywords, and synonyms (Table 1). While the use of a single database means some relevant studies may not have been included, we believe this approach has allowed us to identify the majority of the relevant evidence.

Selection Criteria 

All cohort and case-control studies, and systematic reviews that assessed the inequalities in pregnancy-related health outcomes in Black and South Asian women were considered, regardless of time of publication. Our initial search only included Leicester; however, this did not reveal many relevant studies. Therefore, the search location was expanded to the UK and selected cities that are similar in demographics to Leicester. Only studies undertaken in the UK were considered.

Data Collection

A total of 90 studies were screened independently; titles and abstracts were analyzed to isolate studies that met the criteria. In the initial screening process, 71 studies were rejected, and 18 full-text articles were then reviewed. Although 18 papers were deemed relevant, only 8 were incorporated into this narrative review due to time constraints, and 10 were not included in this review. Eight studies met the criteria and were included in our narrative review. The following studies were not included but may be relevant to the research question: MacLellan et al. [5], Lee and Landau [6], Knight et al. [7], Opondo et al. [8], Kroll et al. [9], Li et al. [10], Puthussery et al. [11], Garcia et al. [12], Petherick et al. [13], and Uphoff et al. [14]. We acknowledge this as a limitation of this narrative review; these studies may contain additional evidence; the omission of these studies could affect the comprehensiveness of the review presented. The studies have been noted above to help guide further research and more comprehensive review efforts.

Quality Appraisal 

No formal quality scoring system was applied given the narrative design of this review; however, the quality of the studies was considered, keeping factors in mind such as how representative the study population was, how reliably exposure and outcome were measured, and whether the study accounted for any confounding factors. These factors were considered to judge how much weight should be given to the findings of the particular study. A summary of the studies and any key limitations is presented within the Results section of this narrative review.

Data Synthesis

A narrative review approach was planned due to the clinical and methodological heterogeneity among the eligible studies, including the variation in study design, population, exposure, definitions, and outcome measures. Findings from the studies are synthesized and presented narratively. 

Results 

Maternal Mortality

The eight studies analyzed showed various findings. Table 2 lists them in detail, along with the key findings and any limitations of individual studies. Overall, the studies showed that there are clear inequalities, not only in maternal mortality and preterm births in Afro-Caribbean and South Asian women but also in other adverse pregnancy-related outcomes. Additionally, being from an ethnic minority is a risk factor for severe pregnancy-related outcomes. Some studies showed that socioeconomic factors do contribute to maternal mortality and preterm births [15]; however, they do not fully explain the differences in outcomes for different ethnicities. None of the studies were able to give a definitive cause, although there were many suggestions, such as genetic factors, microaggressions in healthcare [16], language barriers in first-generation migrants, and a lack of knowledge of the UK healthcare system [17].

**Table 2:** Studies included within this narrative review

Title, date	Population	Study design	Key points	Limitations
Risk factors for progression from severe maternal morbidity to death: a national cohort study, December 2011 [4]	576 women between 2003 and 2009	National cohort analysis	This study conducted an analysis using data from 2003 to 2009 from the Centre for Maternal and Child Enquiries maternal deaths database and the United Kingdom Obstetric Surveillance System database. They found a significant impact on maternal death from increasing age of over 35 years old. There was also an increased risk due to Black ethnicity, with a confidence interval of 1.15-4.92, and unemployment. However, they suggested more research is needed, and evaluation of current services is needed to mitigate the risks of mortality associated with Black ethnicity and lower socioeconomic status [16]. They showed that women who are recent immigrants are more likely to die in pregnancy than those of second generation; no cause has been proven; however, this could be due to language barriers, lack of familiarity with the NHS, cultural norms, or pre-existing conditions not diagnosed within the UK. However, in the Netherlands it has been shown that immigrant women who died in pregnancy were due to a delay in recognizing symptoms and in referral to the GP; a similar situation could be occurring within the UK for recent immigrants of ethnic minority.	The small sample size used may not be representative of the whole UK population. Data were collected from two databases where not all the information they needed was provided.
Inequalities in maternal health: national cohort study of ethnic variation in severe maternal morbidities, March 2009 [15]	686 cases of severe maternal morbidity in all UK hospitals with maternity units	National cohort study	They conducted a national cohort study using the UK Obstetric Surveillance System. They used 686 women between 2005 and February 2006 with severe maternal morbidity in different ethnic groups. Black African women had an increased risk ratio of 2.35, and Black Caribbean women had an increased risk ratio of 2.45, both compared to White women. These figures were adjusted for differences in age, socioeconomic status, smoking, BMI, and parity. Severe maternal morbidity was shown to be significantly more common in non-white women in the UK. This pattern of results is very similar to the ethnic patterns shown with maternal mortality as well. They considered age, socioeconomic status, smoking, BMI, and parity; however, maternal medical factors or factors affecting pregnancy care were not considered and could be impacting this difference alongside ethnicity.	Selection bias may be present, as this study was voluntary; therefore, only certain types of patients in certain areas may volunteer for this study. The data were self-reported; therefore, they may be inconsistent if the definitions were not explicitly written.
A national cohort study and confidential enquiry to investigate ethnic disparities in maternal mortality, January 2022 [16]	1894 women died due to pregnancy-related causes, up to one year after pregnancy, between 2009 and 2018 in the UK	National cohort study	This national cohort study described the characteristics and causes of 1894 maternal deaths between 2009 and 2018 in the UK. The study found that there were no major differences in causes of death between women of different ethnic groups, although this was statistically insignificant. However, they did identify several areas where the maternity care received was biased. Black women were more likely to receive a lack of individualized care, and South Asian women were impacted by microaggressions. Overall, this study concluded that although there were no ethnic inequalities in the causes of maternal mortality, it identified the need for improvement of care given to women, especially Black women, who died during or shortly after pregnancy.	Some records were unavailable, especially of women who died in the late postnatal period, so there may have been some identifiable risk factors that were not considered. Deeper nuances of medical care, including non-verbal communication and negative behaviors, are likely to be undocumented. Causality cannot be ascertained from observation.
Ethnicity and first birth: age, smoking, delivery, gestation, weight and feeding: Scottish Health and Ethnicity Linkage Study, December 2014 [17]	144,344 Scottish women who had a first birth between May 2001 and April 2008	Retrospective cohort study	This study was conducted in Scotland; therefore, the results may not be representative of the whole UK population due to the use of a small sample from a specific location. They used census and health service data to investigate pregnancy outcomes in ethnic groups using a retrospective cohort study. The main findings of this research were that ethnic populations within Scotland had more favorable health behaviors than White Scottish females. Some common health behaviors among White women included smoking, alcohol, and drug abuse; however, the ethnic minority women still gave birth to babies with lower birth weights. This study rules out that health behaviors in different cultures could be the cause of worse pregnancy outcomes.	The study population may not be representative of the whole population, as only Scottish women were considered. As this is a population-based study, it can only determine a correlation rather than provide hard evidence of causality. Recall bias may also be present in a retrospective study, as patients may have trouble recalling events accurately.
Race, prematurity and immaturity, December 2007 [18]	585,291 births were analyzed from the North West Thames obstetric database	Observational study	This research paper compared White Europeans, Black, and South Asian women. They found that Black and South Asian women have a higher incidence of preterm birth and shorter mean gestational length. They also found that Black and South Asian babies have less risk of respiratory distress syndrome. There was a higher survival rate in Black babies than White European babies. They adjusted for socioeconomic factors. They suggested that fetal maturity occurs earlier in gestation in Black and South Asian babies, as shown by the decreased risk of respiratory distress syndrome. They suggested preterm births may be increased within the Black and South Asian population; however, there is decreased risk of respiratory distress syndrome compared with White European babies.	The data are collected from the North West Thames obstetric database, which requires self-reporting and self-identification; therefore, there is potential for misclassification that could affect the results. The data were obtained only from one specific area; therefore, they may not be generalized to the whole population due to different environmental factors within a specific area.
Adverse pregnancy outcomes attributable to socioeconomic and ethnic inequalities in England: a national cohort study, November 2021 [19]	981 women who delivered singletons with a recorded gestation period between 24 and 42 weeks in 132 NHS hospitals in England from 2015 to 2017	National cohort study	This national cohort study analyzed birth records from 132 NHS hospitals for babies born between 24 and 42 weeks. The criteria included stillborn, preterm, and fetal growth restriction birthweight in England, and these outcomes were compared by socioeconomic status and ethnicity. For preterm births, the findings were worse for Black women and South Asian women compared with White women, although the differences were small. The risk ranged from 6% in white women, 6.6% in Black women, and 6.5% in South Asian women. Interestingly, the study found that 12% of stillbirths and 1% of preterm births could have been prevented if Black and South Asian women had the same risks as White women. Overall, the study shows that adverse pregnancy outcomes may be attributed to the combined effect of multiple maternal characteristics. The adverse pregnancy outcomes may be linked to a wider pattern of poorer health circumstances in Black and South Asian women.	The study found no major difference in the risk of preterm births, as the percentages were very similar. However, the significance must be calculated, as the population of Black and South Asian women was smaller, so a smaller percentage would be expected. The study used an aggregate area-based measure to capture deprivation, which may have led to misclassification of some women; this could lead to underestimation of the effect of socioeconomic status.
The risk of preterm delivery in women from different ethnic groups, August 2002 [20]	All North Birmingham women delivering singletons between 1994 and 1997 inclusive from the child health record system	Cross-sectional study	The aim of this paper was to examine whether variables explain the increased risk of preterm delivery in ethnic groups. They conducted a cross-sectional study in North Birmingham. Birmingham has similar demographics to Leicester, with a high ethnic minority. The OR for Afro-Caribbean women having delivery before 37 weeks was 1.22, with a CI of 1.07-1.41. Before 34 weeks, there is an OR of 1.32 with a CI of 0.84-2.06; therefore, before 34 weeks, the results may be due to chance. For African women, there was no increased risk shown at 37 weeks; however, there was an adjusted OR of 4.1 and a CI of 1.66-10.16 for delivery under 28 weeks. They adjusted the odds ratio for deprivation and marital status, as these did increase the risk of preterm delivery, showing a correlation between deprivation and preterm delivery in all ethnic minorities except Asians. They stated that half of the excess risk in Afro-Caribbeans was accounted for by the two risk factors, deprivation and marital status. However, the increased risk of preterm delivery shown in Africans and Afro-Caribbeans could be explained by complex social factors and maybe by early maturity of the fetoplacental unit. Other suggestions could be variations in vaginal flora, which could be different across ethnic groups due to genetic or cultural differences.	Although the study included other potential confounding risk factors for preterm delivery, there was no data on smoking. Ethnic coding varied across hospitals, so they collapsed the more detailed, smaller ethnic groups into broader groups: White, Asian, Afro-Caribbean, African, Mixed, and other, thus conflating different cultures, which could introduce confounding.
The role of social risk factors and engagement with maternity services in ethnic disparities in maternal mortality: a retrospective case note review, October 2022 [21]	196 women who died during or up to a year after pregnancy, in the UK, between 2015 and 2017	Retrospective systematic case notes review	This study consisted of a retrospective review of medical notes of 219 women who died between 2015 and 2017 in the UK. The study aimed to explore whether social risk factors or access to maternity services could explain the disparities in maternal mortality. They identified both pre-existing physical comorbidities and complex social risk factors that were faced by women regardless of ethnicity, including domestic violence, substance abuse, insecure housing, mental health issues and learning disabilities, among others. The women were grouped by ethnicity: White British, White European, other than White, with Black African and Pakistani women being the most common ethnicities in the latter group. Frequencies of physical comorbidities and complex social risk factors were calculated for the women who died. The study found that three or more complex social factors were present in 36.7% of white British women, compared with approximately 12% among those who were other than White. On the other hand, 15.5% of other than White women had three or more physical comorbidities, as opposed to 3.3% in White British women. In addition, the study identified 17 women who required an interpreter and did not receive one throughout their maternity care. Overall, this study concludes that the differences in access to maternity services and social risk factors do not explain the ethnic disparities in maternal mortality.	The sample size was relatively small; therefore, no statistical comparison was carried out. Using the category “other than White” is an oversimplification and masks the diversity of ethnic groups.

Multiple studies showed that being of ethnic minority was a risk factor for adverse pregnancy-related outcomes. One study used 686 women between 2005 and 2006 with severe maternal morbidity in different ethnic groups. Black African women had an increased risk ratio of 2.35 and Black Caribbean women of 2.45 when compared with White women [15]. These figures were significant after being adjusted for differences in age, socioeconomic status, smoking, BMI, and parity. Severe maternal morbidity was shown to be significantly more common in non-White women in the UK; this pattern of results is very similar to the ethnic patterns shown with maternal mortality as well [15]. This massive increased risk ratio shows the inequalities present in ethnic minorities compared with their Caucasian counterparts. The common causes thought to affect these outcomes were considered, and risk ratios were adjusted accordingly, such as BMI, smoking, and socioeconomic status. No clear explanation was identified. 

Preterm Birth

One further study reported Black and South Asian origin that showed better health behaviors than those of Caucasian background [17]. The health behaviors included smoking, alcohol, and drug abuse. This study highlights that the inequalities observed in ethnic minorities cannot be explained by worse health behaviors [17].

One study reported that although Black and South Asian women had a higher incidence of preterm birth, this was associated with a shorter gestational length and a lower risk of respiratory distress syndrome in babies compared with White European babies, raising the possibility that increased preterm birth risk in these groups may partly reflect a difference in the typical gestational timing rather than solely being an adverse outcome [18]. This raises a question as to whether current definitions of prematurity account for any possible ethnic variation in fetal maturation, although evidence of this is limited. 

A further study that was included analyzed birth records from 132 NHS hospitals and outlined that the increase in preterm birth in ethnic minorities was relatively small, with an increased risk of 0.6% in Black women and a 0.5% increase in South Asian Women [19]. However, it was estimated that 12% of stillbirths and 1% of preterm births could have been prevented if these ethnic minorities had the same risks as White women. This reflects that adverse pregnancy outcomes cannot be explained by a single factor, and these outcomes are preventable, meaning action should be taken to reduce the risk of adverse outcomes. However, this study's findings were notably smaller in magnitude than those reported elsewhere in the literature reviewed here. 

Health Behaviors 

A study conducted in Birmingham showed a correlation between deprivation and socioeconomic status with preterm delivery, suggesting these are key factors in increasing the risk of preterm delivery in Afro-Caribbean Women. However, it was acknowledged that these two factors, along with marital status, cannot fully explain the excess risk in Afro-Caribbean Women [20]. Many other suggestions were given by this paper to explain the excess risk, such as complex social factors, possible early maturity of the fetoplacental unit, and variations in vaginal flora; however, these are only suggestions and have not been proven [20]. 

Summary

Multiple study designs and populations are included in this narrative review; despite this variety, these studies have shown a consistent increased risk in maternal mortality and preterm birth in ethnic minority. These included studies vary in methodological quality, as evidenced by the limitations listed in Table 2. The majority of included studies recommended that further research is needed to further understand the root causes of these inequalities.

Discussion

Interpretation of Findings 

This narrative review shows consistent evidence across multiple UK studies of clear, observed inequalities present in maternal mortality and pre-term birth in ethnic minorities. This finding, spanning across multiple studies, is unlikely to be solely due to chance and represents an important public health concern.

Many of these studies demonstrated multiple factors that affect preterm birth and risk of maternal mortality, including ethnicity, complex social factors, socioeconomic factors, marital status, and deprivation. Health behaviors were shown to be better in ethnic minorities and worse in Caucasian women, with Caucasian women more likely to have characteristics such as maternal smoking, mental health issues, and alcohol and substance abuse. Therefore, health behaviors are not likely to be the cause of the difference between the two outcomes [17]. These findings challenge the assumption that lifestyle factors can explain the ethnic inequalities. This also suggests that we cannot resolve these inequalities by simply focusing on health behavior measures such as encouraging people to stop smoking. Broader structural and healthcare-related factors are more likely to be contributing to these inequalities.

All the published research showed that there is a difference in premature delivery and maternal mortality within ethnic minorities. However, there was little evidence as to a reason for these ethnic disparities. The published studies could only offer suggestions; however, no single explanatory factor emerged. These suggestions included genetic variations, early maturity, and multiple negative circumstances [19], causing increased risk of preterm birth [20]. Other suggested factors included language barriers, cultural differences, and unfamiliarity with the UK healthcare system. This could be a plausible reason as to why there was an increased maternal mortality; however, these explanations would only apply to first-generation migrants and not second-generation ethnic minorities who were born within the UK and who still have an increased risk of premature birth and maternal mortality [17]. There was also a suggestion that Black and South Asian women have a negative experience of maternity care [17] and a lack of interpreter services when required [21]. This would understandably lead to negative maternal outcomes; however, this explanation again cannot be applied to second-generation ethnic minorities. This suggests that migration-related factors alone are not adequate explanations. Other factors such as implicit bias, discrimination, and microaggressions may warrant further investigation in the role they play in these inequalities. Overall, there does not seem to be one core reason to explain these inequalities; however, a complex combination of biological, social, and healthcare factors is the likely cause.

Comparison With Existing Literature 

This narrative review focuses solely on UK-based evidence, aiming to explore its applicability to Leicester and Leicestershire. Comparison with international literature on ethnic disparities in maternal outcomes was therefore outside the scope of this narrative review. This is a limitation, as international research, particularly from countries such as the USA, is likely more extensively studied and could therefore offer additional insight into the contributing factors and mechanisms that cause these inequalities. These additional mechanisms may not yet have been explored within the UK. Further reviews may benefit from including wider non-UK research and identifying factors that could be applied to the UK healthcare model.

Strengths and Limitations

One strength worth highlighting within this narrative review is the consistency of findings across the multiple UK-based studies. The studies included used multiple populations and designs, further strengthening the fact that the identified disparities are genuine and not artifacts of a single study. However, it must be acknowledged that many of these studies are observational and are unable to establish causality. Further limitations to each specific study can be seen in Table 2 shown in the Results section. Some studies have adjusted for confounding variables; however, many studies have been unable to definitively list factors with a direct link to the cause of this inequality.

Overall, the studies included varied in methodological quality. Table 2 in Results highlights the key limitations of each study, with the strongest evidence being a large national cohort study where confounding variables have been adjusted, while other studies included used smaller local samples. No formal quantitative methodological assessment was conducted within this narrative review; however, the quality of the studies was acknowledged.

One limitation of this narrative review was the inability to identify any studies relating directly to our area of interest, that is, Leicester. All the studies included within this search were conducted within the UK, including different cities that have similar demographics to Leicester, such as Bradford. However, as discussed in the Introduction, Leicester has a highly diverse population with a high ethnic minority. This unique demographic may further increase the observed ethnic inequality compared with other parts of the UK. Due to this unique demographic, some reasons given in other parts of the UK may not be applicable or as relevant to Leicester, such as language barriers, as these services are readily available due to a high ethnic minority. These findings should therefore be considered a guide to, rather than direct evidence from, Leicester and Leicestershire. This identifies the need for more specific research within Leicester into the reasons these inequalities exist, investigating whether they are intensified and whether population-specific factors contribute.

A key limitation of this narrative review was the exclusion of 10 eligible studies, as mentioned in the Methods section of this review. This exclusion may have introduced selection bias and means evidence here should not be considered fully comprehensive. These studies have been included within the references section for future reviews to provide a more complete picture.

*Implications for Practice and Future Research* 

The inability of multiple studies to list direct causes highlights the complex combination of factors leading to these proven inequalities and the limitations of current evidence available. Efforts to address these inequalities with a single focus intervention such as health behavior campaigns are likely to be unsuccessful. These findings instead show the need for further research and investigation into structural and healthcare-related contributors, including, as mentioned before, implicit bias, discrimination, and accessibility of maternity care. Research within Leicester and Leicestershire is also needed to determine whether these inequalities observed in other parts of the UK are intensified within this area due to its unique demographics, and to investigate whether any further contributing factors specific to this unique population are present.

Recommendations 

We would recommend a study consisting of interviews and focus groups with midwives and antenatal care staff to gain a better understanding of the nuances of maternity care within Leicester and Leicestershire. Focus groups and discussions with Black and South Asian women would also be helpful in triangulating the causes of the disparity in maternal mortality and premature births. Both qualitative and quantitative studies are required specifically for the Leicester demographic.

This review has identified the inequalities that women from ethnic minorities face in the UK, and the cause of such inequalities is unlikely to be individual, clinical, or lifestyle factors. Based on the patterns observed, it is plausible that factors such as access to maternity services and variation in the quality of services could be contributing to these inequalities. However, this remains a hypothesis arising from the review rather than a direct conclusion supported by the included evidence, as no study directly evaluated the impact that access or bias has on these inequalities. Further research, including the qualitative and quantitative research mentioned above, is needed to test this hypothesis before any policy changes and public health efforts can be made. Should this research confirm this hypothesis, public health efforts can be appropriately directed toward improving equitable access to maternity services and the consistency of care provided across populations.

## Conclusions

Overall, this review of previous literature suggests that there is an increased risk of premature birth and maternal mortality among ethnic minorities. One study attributed this increased risk of premature births to fetal maturity occurring earlier in gestation in ethnic minority women. No study identified a definitive cause of the increased risk. Although multiple studies have mentioned explanations such as language barriers, variations in maternity care, biological factors, social factors, and socioeconomic status, no single explanation was consistently supported across the included studies. Based on the evidence included within this narrative review, the increased risk is most likely due to multiple complex factors that affect pregnancy outcomes, though this remains an interpretation drawn from a limited evidence base rather than a definitive conclusion.

More research is needed, particularly in Leicester and Leicestershire due to its unique demographics, to investigate the specific underlying reasons as to why Black and South Asian women have increased risk of premature delivery and maternal mortality. The findings of this narrative review suggest that solely modifying individual behaviors, such as reducing smoking, is unlikely to improve ethnic inequalities. Rather, multiple interventions, such as improving access to maternity services, increasing the availability of interpreter services, and reducing microaggressions within the NHS, may be worth further investigation as potential contributors. As no included study directly tested these factors, this should be considered a hypothesis for future research rather than a confirmed basis for intervention.
